# Microbes in food biotechnology: An annotated selection of World Wide Web sites relevant to the topics in *microbial biotechnology*

**DOI:** 10.1111/1751-7915.13542

**Published:** 2020-02-13

**Authors:** Lawrence P. Wackett

**Affiliations:** ^1^ Department of Biochemistry, Molecular Biology & Biophysics BioTechnology Institute University of Minnesota St. Paul MN 55108 USA

## Microbial food biotechnology compilation

https://www.biomedcentral.com/collections/food-biotech

This page hosts a repository of links to journal articles in *Microbial Cell Factories* related to different aspects food biotechnology.

## Introduction to the microbiology of food

https://aggie-horticulture.tamu.edu/food-technology/food-processing-entrepreneurs/microbiology-of-food/

This webpage provides a good, broad overview of food microbiology.

## Biotechnology in food for developing countries

http://www.fao.org/biotech/C11doc.htm

It is indicated here that one‐third of the human diet worldwide derives from fermented foods. Given that these foods are often indigenous and linked to culture, these are important considerations as food biotechnology becomes more prevalent in developing countries.

## Microorganisms in food and beverage preparation

https://en.wikipedia.org/wiki/List_of_microorganisms_used_in_food_and_beverage_preparation

This page contains a very long list of genera and species of bacteria involved in food and beverage production and indicates the food they they produce.

## Microorganisms in food

https://www.wikilectures.eu/w/Micro-organisms_in_Foods

This page briefly covers microbes involved with foods in different ways, as: human pathogens, spoilage agents or in production or processing.

## Microbial enzymes in the food industry

https://www.ncbi.nlm.nih.gov/pmc/articles/PMC5956270/

Enzymes are widely used in the food industry. Some have their origins from microbial systems. Others are originally from plants or animals but are biosynthesized in microbes recombinantly.

## Microbial interactions in food fermentations

https://www.annualreviews.org/doi/abs/10.1146/annurev-food-022811-101219

This is a helpful review article althought its content may only be available through university libraries.

## Laboratory of food biotechnology

https://fbt.ethz.ch/research.html

This laboratory studies food biotchnology and complementary gut microbial metabolism, with the goal of oprimizing nutrition and human health.

## Bio organization: On food additives

https://archive.bio.org/articles/biotechnologys-impact-food-ingredients

This website is put out by the Biotechnology Innovation Organization, or Bio. It briefly deals with major contemporary issues in food biotechnology.

## Microbes help make the coffee

https://www.sciencedaily.com/releases/2019/02/190201130624.htm

Wet processing of coffee utilizes a microbial fermentative step. Lactic acid bacteria play a role, as do other bacteria, in that process.

## Food biotechnology markets

https://www.globenewswire.com/news-release/2019/05/23/1841415/0/en/Food-Biotechnology-Market-value-to-hit-45-Billion-by-2025-Global-Market-Insights-Inc.html

This business report concludes that the food biotechnology market is growing rapidly and is predicted to reach $45 billion by the year 2025.

## Recent food microbiology research and reviews

https://www.nature.com/subjects/food-microbiology

This page by *Nature* publishers is a compendium of recent peer‐reviewed publications in *Nature* journals related to food microbiology.

## Food biotechnology: Open access articles

https://network.bepress.com/life-sciences/food-science/food-biotechnology/

This page of the Food Biotechnology Commons links to numerous open source articles related to food biotechnology.

## Biotechnology and food

https://www.biotech.wisc.edu/docs/default-source/outreach-documents/biotechandfood.pdf?sfvrsn=dd91ec28_2

This extension report focuses on food and agricultural biotechnology.

## The GRAS process for microbial enzymes

https://www.liebertpub.com/doi/full/10.1089/ind.2016.0011

GRAS stands for “Generally Regarded As Safe.” Enzymes made from food safe organisms would be safe to use for food applications.

## History of *Lactobacillus acidophilus*

https://academic.oup.com/femsle/article/349/2/77/533643

*Lactobacillus acidophilus* was originally isolated from the human intestinal tract and is today an important strain for yogurt and other dairy products.

## *Lactobacillus acidophilus* genomes

https://www.ncbi.nlm.nih.gov/genome/genomes/1099?

This page of the National Center for Biotechnology Information (NCBI) links to the genome sequences and related data for many *Lactobacillus acidophilus* strains.

